# Substrate Promiscuity of *N*-Acetylhexosamine 1-Kinases

**DOI:** 10.3390/molecules16086396

**Published:** 2011-07-28

**Authors:** Yanhong Li, Hai Yu, Yi Chen, Kam Lau, Li Cai, Hongzhi Cao, Vinod Kumar Tiwari, Jingyao Qu, Vireak Thon, Peng George Wang, Xi Chen

**Affiliations:** 1 Department of Chemistry, University of California, One Shields Avenue, Davis, CA 95616, USA; Email: yhzli@ucdavis.edu (Y.L.); hyu@ucdavis.edu (H.Y.); syichen@ucdavis.edu (Y.C.); edlau@ucdavis.edu (K.L.); jqu@ucdavis.edu (J.Q.); vthon@ucdavis.edu (V.T.); 2 Departments of Biochemistry and Chemistry, Ohio State University, Columbus, OH 43210, USA; Email: lcai@chemistry.ohio-state.edu (L.C.); wang.892@osu.edu (P.G.W.)

**Keywords:** *N*-acetylgalactosamine, *N*-acetylglucosamine, *N*-acetylhexosamine 1-kinase, mannose, substrate specificity

## Abstract

*N*-Acetylhexosamine 1-kinase (NahK) catalyzes the direct addition of a phosphate from adenosine 5'-triphosphate (ATP) to the anomeric position of *N*-acetylhexosamine and shows similar activity towards *N*-acetylglucosamine (GlcNAc) and *N*-acetylgalactosamine (GalNAc). Herein we report the cloning, characterization, and substrate specificity studies of two NahKs from *Bifidobacterium infantis* ATCC15697 and *Bifidobacterium longum* ATCC55813, respectively. A new capillary electrophoresis assay method has been developed for enzyme activity assays. Both enzymes have a good expression level in *E. coli* (180–185 mg/L culture) and can tolerate diverse modifications at C2 of GlcNAc and GalNAc. Various GlcNAc derivatives with C6, both C2 and C6, as well as both C2 and C3 modifications are tolerable substrates for the newly cloned NahKs. Quite interestingly, despite of their low activities toward glucose and galactose, the activities of both NahKs are much higher for mannose and some of its C2, C4, and C6 derivatives. These NahKs are excellent catalysts for enzymatic and chemoenzymatic synthesis of carbohydrates.

## 1. Introduction

*N*-Acetylglucosamine (GlcNAc) and *N*-acetylgalactosamine (GalNAc) are important mono-saccharides broadly distributed in Nature. GlcNAc plays an important role in plant organogenesis and invertebrate embryogenesis [1]. It is an essential component of protein *N*-glycans and some important polysaccharides including chitin (the second most abundant carbohydrate after cellulose) [2,3], bacterial cell wall [4], and some glycosaminoglycans such as hyaluronic acid, keratan sulfate, and heparan sulfate/heparin [5,6,7]. It also exists in many *O*-glycans. In addition, glycoproteins modified with *O*-GlcNAc monosaccharide have been increasingly identified [8,9]. In comparison, GalNAc is an essential component of protein *O*-glycans and some glycosaminoglycans such as chondroitin sulfate and dermatan sulfate [5]. It also exists in many gangliosides. Therefore, it is in an urgent need to develop high efficient processes for producing GlcNAc and GalNAc-containing carbohydrates and glycoconjugates.

In Nature, the biosynthesis of GlcNAc and GalNAc-containing oligosaccharides and glycoconjugates is carried out by corresponding glycosyltransferases which require sugar nucleotide donors such as uridine 5'-diphospho-GlcNAc (UDP-GlcNAc) and uridine 5'-diphospho-GalNAc (UDP-GalNAc). For *in vitro* enzymatic synthesis of these compounds, *in situ* generation of sugar nucleotides is a common practice to reduce the synthetic cost. The simplest route for enzymatic synthesis of both UDP-*N*-acetylhexosamines (UDP-HexNAc) and their derivatives is the combined use of an *N*-acetylhexosamine 1-kinase (NahK) [10,11,12] and an *N*-acetylglucosamine-1-phophate uridyltransferase (GlmU [13,14] or AGX1 [15]).

NahK (EC 2.7.1.162) catalyzes the direct addition of a phosphate from adenosine 5'-triphophate (ATP) to the anomeric position of *N*-acetylhexosamine for the formation of *N*-acetylhexosamine-1-phosphate and adenosine 5'-diphophate (ADP). The only characterized NahK to date is encoded by the *lnpB* gene in the *lnpABCD* operon of *Bifidobacterium longum* JCM1217 [10]. Herein we report the cloning and characterization of two new NahKs from *Bifidobacterium infantis* (ATCC15697) and *Bifidobacterium longum* (ATCC55813), respectively. A new capillary electrophoresis-based assay method has been developed for biochemical characterization of NahKs. We found that in addition to previously reported NahK substrates, various GlcNAc derivatives including those with C2-azido, C6-azido, and 6-*O*-sulfate groups are tolerable substrates for the newly cloned NahKs. In addition, despite of their low activities toward glucose and galactose, the activities of both NahKs are much higher for mannose and some of its C2, C4, and C6 derivatives including 2-deoxymannose or 2-deoxyglucose.

## 2. Results and Discussion

### 2.1. Cloning, Expression, and Purification

**Figure 1:**
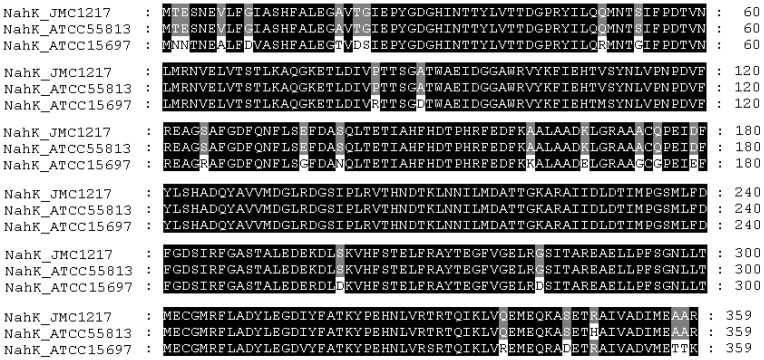
Sequence alignment of NahK_JCM1217 (GenBank accession no. BAF73925), NahK_ATCC55813, and NahK_ATCC15697.

NahKs from *Bifidobacterium infantis* ATCC#15697 (NahK_ATCC15697) and *Bifidobacterium longum *ATCC#55813 (NahK_ATCC55813) were each cloned as a C-His_6_-tagged fusion protein in a pET22b(+) vector. Sequence alignment (Figure 1) indicates that NahK_ATCC55813 is almost identical to the NahK from *Bifidobacterium longum *JCM1217 (NahK_JCM1217, GenBank accession no. BAF73925) except for a single amino acid difference R348H (R is in NahK_JCM1217). In comparison, NahK_ATCC15697 shares 90% amino acid sequence identity with NahK_JCM1217.

**Figure 2:**
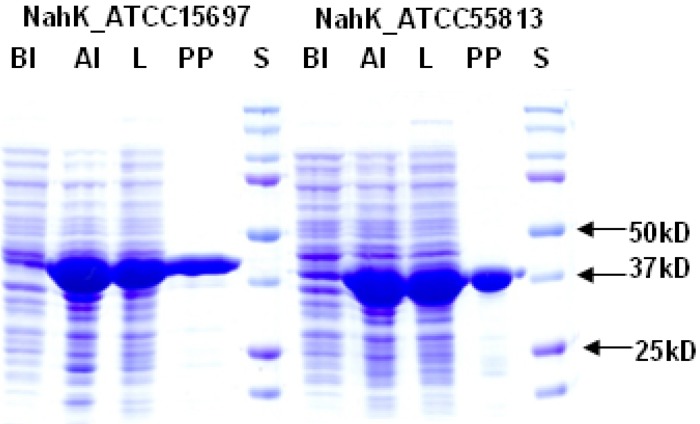
SDS-PAGE analysis of NahK_ATCC15697 and NahK_ATCC55813. Lanes: BI, whole cell extract before induction; AI, whole cell extract after induction; L, lysate; PP, purified protein; S, Bio-Rad Precision Plus Protein Markers (10–250 kDa).

Both NahKs were expressed by induction with 0.1 mM of isopropyl-1-thio-β-D-galactopyranoside (IPTG) followed by incubation at 20 °C for 24 h with vigorous shaking (250 rpm). Up to 180 mg and 185 mg of Ni^2+^-column purified NahK_ATCC15697 and NahK_ATCC55813, respectively, could be obtained from one liter of *E. coli* culture. SDS-PAGE analysis (Figure 2) shows that both purified proteins migrated to around 41 kDa, matching well to the calculated molecular weights of the translated His_6_-tagged fusion proteins of 41.4 and 40.9 kDa for NahK_ATCC15697 and NahK_ATCC55813, respectively.

### 2.2. Capillary Electrophoresis (CE) Assays

Based on the detection of ADP and ATP in the reaction mixture by a UV detector, a capillary electrophoresis-based method was developed to directly measure the formation of ADP and *N*-acetylhexosamine-1-phosphate from ATP and *N*-acetylhexosamine for characterizing the activities of NahKs. Both ATP and ADP gave absorbance at 254 nm with equal signal responses.

### 2.3. pH Profile

**Figure 3:**
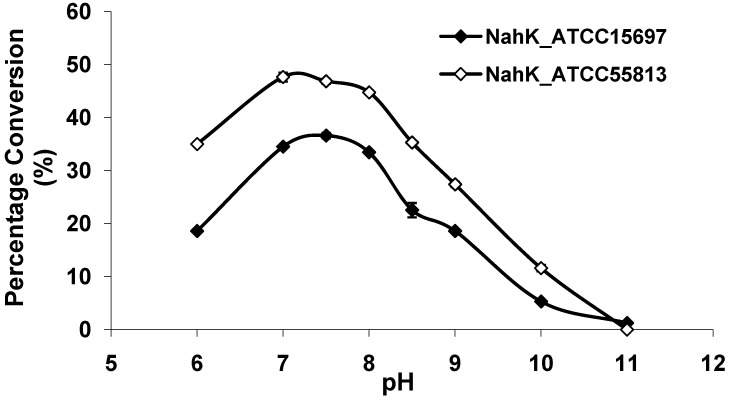
pH profiles of NahK_ATCC15697 (♦, filled diamond) and NahK_ATCC55813 (à, open diamond). Buffers used: MES, pH 6.0; Tris-HCl, pH 7.0–9.0; CAPS, pH 10.0–11.0.

As shown in Figure 3, both NahKs are highly active in a pH range of 7.0–8.0 with slight variations. The activities of both NahKs drop quickly with either decrease of the pH to below 7.0 or an increase of the pH to more than 8.0. About 50% of the optimal activity was observed at pH 6.0 and pH 8.5 for NahK_ATCC15697. In comparison, about 70% of the optimal activity was observed at pH 6.0 and pH 8.5 for NahK_ATCC55813. The pH optima of these two enzymes are slight different from that (pH 8.5) of NahK_JCM1217 [10]. Overall, the activity of NahK_ATCC55813 is higher than that of NahK_ATCC15697 in the pH range of 6.0–10.0 when GlcNA was used as the substrate and the same molar concentrations of the enzymes were used.

### 2.4. Effect of MgCl_2_

**Figure 4:**
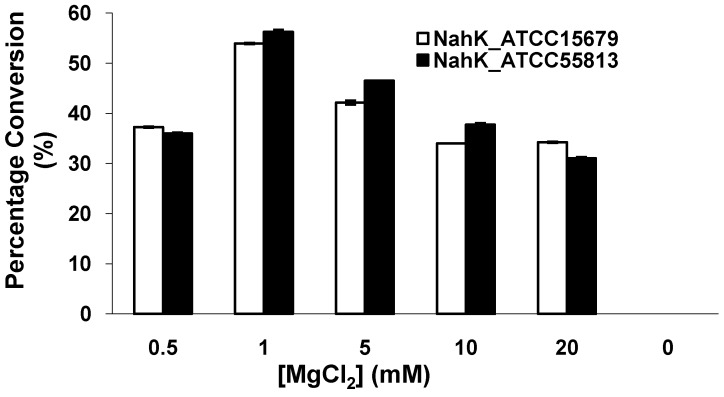
The effect of MgCl_2_ on the activities of NahKs.

Similar to NahK_JCM1217 [10] and other kinases, both NahK_ATCC15697 and NahK_ATCC55813 require a divalent metal ion for activity. As shown in Figure 4, the optimal concentration of Mg^2+^ was determined to be 1 mM. The activities of both NahKs in the presence of 0.5 mM of Mg^2+^ were about two thirds of those in the presence of 1.0 mM of Mg^2+^. Increasing the concentration of Mg^2+^ from 1 mM to 20 mM caused a slight decrease of the activities of both NahKs.

### 2.5. Kinetics

The apparent kinetic parameters shown in Table 1 indicate that the activities of two NahKs are close, with NahK_ATCC55813 having 16% or 39% higher activity than NahK_ATCC15697 when GlcNAc or GalNAc was used as the substrate in the presence of ATP. Overall, GlcNAc is a more efficient (3.1-fold for NahK_ATCC15697 and 2.6-fold for NahK_ATCC55813) substrate than GalNAc for both NahKs due to relatively lower *K_m_* values and higher (~2-fold) *k_cat_* values obtained when GlcNAc was used. Using ATP and GlcNAc as the substrates, the *K_m_* values of ATP (0.10 ± 0.03 mM and 0.11 ± 0.03 mM) and GlcNAc (0.06 ± 0.01 mM) for both NahKs are lower than those for NahK_JCM1217 (0.172 mM for ATP and 0.118 mM for GlcNAc) determined by high performance ion chromatography (HPIC) with a pulsed amperometric detector (DX500, Dionex Corporation, Sunnyvale, CA, USA) using a Dionex CarboPac PA1 column (4 mm × 250 mm) [10]. The discrepancies of the parameters may be due to the differences in the assay conditions used.

**Table 1:** Apparent kinetic parameters of NahKs.

Enzymes	NahK1_ATCC15697			NahK_ATCC55813		
Substrate	*K_m_* (mM)	*k_cat_* (s^−1^)	*k*_cat_/*K_m_* (s^−1^ mM^−1^)	*K_m_* (mM)	*k_cat_* (s^−1^)	*k*_cat_/*K_m_* (s^−1^ mM^−1^)
ATP ^a^	0.10 ± 0.03	1.1 ± 0.1	11.0	0.11 ± 0.03	1.3 ± 0.1	11.8
GlcNAc	0.06 ± 0.01	0.95 ± 0.01	15.8	0.06 ± 0.01	1.1 ± 0.1	18.3
ATP ^b^	0.08 ± 0.03	0.38 ± 0.02	4.8	0.06 ± 0.02	0.48 ± 0.03	8.0
GalNAc	0.09 ± 0.05	0.46 ± 0.07	5.1	0.08 ± 0.03	0.57 ± 0.04	7.1

^a^ The other substrate is GlcNAc; ^b^ The other substrate is GalNAc.

### 2.6. Substrate Specificity

The substrate specificity studies using GlcNAc, GalNAc, and their derivatives (Table 2) indicate that both NahKs exhibit promiscuous substrate specificity and have comparable levels of activity toward GlcNAc and GalNAc derivatives. Compared to NahK_ATCC15697, NahK_ATCC55813 is more reactive towards non-modified GlcNAc (**1**), GalNAc (**11**), and some of their C2-modified derivatives with an *N*-trifluoroacetyl (GlcNTFA **2** and GalNTFA **12**), an *N*-azidoacetyl group (GlcNAcN_3_ **3** and GalNAcN_3_ **13**), or an *N*-butanoyl group (GlcNBu **4** and GalNBu **14**). Nevertheless, NahK_ATCC15697 is more reactive than NahK_ATCC55813 for some of C2-modified GlcNAc and GalNAc derivatives such as those with a bulky *N*-benzoyl group (GlcNBz **5** and GalNBz **15**) and a C2-azido group (GlcN_3_ **6** and GalN_3_ **16**). NahK_ATCC15697 is also more reactive towards 2-amino-2-deoxy-glucose (GlcNH_2_ **7**), 2-*N*-sulfo-glucose (GlcNS **8**), as well as C6-modified GlcNAc derivatives such as 6-deoxy-GlcNAc (GlcNAc6Me **9**), 6-azido-6-deoxy-GlcNAc (GlcNAc6N_3_ **10**), and 6-*O*-sulfo-GlcNAc (GlcNAc6S **17**). Both C2 and C6-modified derivatives GlcNAc such as 6-*O*-sulfo-*N*-trifluoroacetyl glucosamine (GlcNTFA6S **18**) and 6-*O*-sulfo-2-azido-2-deoxy glucose (GlcN_3_ **19**) as well as both C2 and C3-modified GlcNAc derivative 3-*O*-sulfo-2-azido-2-deoxy glucose (GlcN_3_3S **20**) are poor but acceptable substrates for both enzymes. Overall, some of the C2-modified GlcNAc and GalNAc (**1–5** and **11–14**) are relatively good substrates for both NahKs with yields varied from 5.2%–42.3% in a 10 min reaction containing 0.75 μM of enzyme. In comparison, other C2-modified GlcNAc and GalNAc (**6–8**, **15**, **16**), C6- (**9**, **10**, **17**), both C2- and C6- (**18**, **19**), as well as both C2- and C3-modified GlcNAc (20) derivatives are poor but tolerable substrates for both NahKs and the assays have to be carried out for a longer reaction time (30 min) with a 20-fold higher concentration (15 μM) of enzyme.

Among twenty compounds of GlcNAc, GalNAc and their derivatives tested, compounds **1**, **3–5**, **9–11**, **13–15** have been reported before as suitable substrates for NahK_JCM1217 [11,12], while other compounds including **2**, **6–8**, **12**, and **16–20** are newly identified substrates for NahKs. It is worth to note that some of these compounds have negatively charged *O*-sulfate group at different positions of GlcNAc or its derivatives.

Quite interestingly, the substrate specificity studies using glucose (Glc **21**), galactose (Gal **28**), mannose (Man **23**), *N*-acetylmannosamine (ManNAc **29**), and derivatives of mannose and ManNAc (Table 3) indicate that while both Glc (**21**) and Gal (**28**) are poor substrates for both NahKs, 2-deoxy glucose (2-deoxyGlc **22**) or 2-deoxymannose is a better substrate. In addition, mannose (**23**), its 2-fluoro- (2F-Man **24**) and 2-azido- (2N_3_-Man **26**) derivatives, as well as its 4-deoxy (4-deoxyMan 27) derivative are relatively good substrates. In comparison, 2-methyl modification of mannose (2Me-Man 25) decreases its tolerance as the substrate for both NahKs. Quite surprisingly, while ManNAc (29) and some of its C2 derivatives (**30–32**) are poor substrates for the NahKs, *N*-azidoacetylmannosamine (ManNAcN_3_ **33**, a C2-derivative of ManNAc) and its C6-derivative *N*-acetyl-6-*O*-methylmannosamine (ManNAc6OMe **34**) are better substrates for both NahKs. Overall, except for 2-fluoro-mannose (2F-Man **24**), NahK_ATCC15697 shows higher activity than NahK_ATCC55813 for mannose, ManNAc, and their derivatives.

**Table 2:** Substrate specificity of NahKs using GlcNAc, GalNAc, and their derivatives.

Substrates	Percentage Conversion (%)				Substrates	Percentage Conversion (%)			
	NahK_ATCC15697		NahK_ATCC55813			NahK_ATCC15697		NahK_ATCC55813	
	^a^ 0.75 μM	^b^ 15 μM	^a^ 0.75 μM	^b^ 15 μM		^a^ 0.75 μM	^b^ 15 μM	^a^ 0.75 μM	^b^ 15 μM
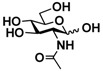 **1** GlcNAc	35.4 ± 0.1	NA	42.3 ± 0.2	NA	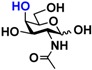 **11** GalNAc	12.5 ± 0.1	NA	19.9 ± 0.1	NA
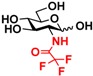 **2** GlcNTFA	10.7 ± 0.9	NA	16.2 ± 0.9	NA	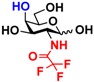 **12** GalNTFA	11.2 ± 1.6	NA	21.8 ± 0.2	NA
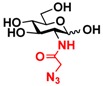 **3** GlcNAcN_3_	11.5 ± 1.0	NA	22.8 ± 0.4	NA	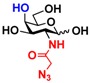 **13** GalNAcN_3_	9.9 ± 0.6	NA	21.0 ± 1.2	NA
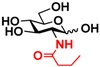 **4** GlcNBu	20.9 ± 0.6	NA	35.0 ± 2.0	NA	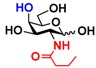 **14** GalNBu	12.1 ± 0.3	NA	24.0 ± 0.1	NA	
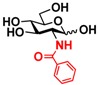 **5** GlcNBz	10.3 ± 0.4	NA	5.2 ± 0.2	NA	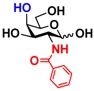 **15** GalNBz	0	62.2 ± 1.0	0	51.9 ± 0.5	
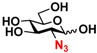 **6** GlcN_3_	0	14.5 ± 0.1	0	7.0 ± 0.1	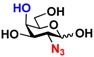 **16** GalN_3_	0	7.6 ± 0.1	0	4.3 ± 0.1	
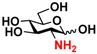 **7** GlcNH_2_	0	15.0 ± 0.1	0	8.4 ± 0.1	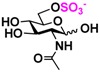 **17** GlcNAc6S	0	11.7 ± 0.2	0	6.6 ± 0.1
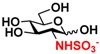 **8** GlcNS	0	6.4 ± 0.2	0	4.0 ± 0.1	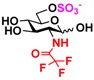 **18** GlcNTFA6S	0	7.2 ± 0.1	0	3.3 ± 0.2
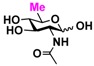 **9** GlcNAc6Me	4.4 ± 1.2	41.8 ± 0.3	2.1 ± 0.2	36.3 ± 0.3	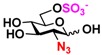 **19** GlcN_3_6S	0	6.9 ± 0.1	0	4.4 ± 0.1
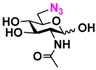 **10** GlcNAc6N_3_	0	37.2 ± 0.5	0	23.4 ± 0.1	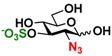 **20** GlcN_3_3S	0	4.9 ± 0.1	0	3.9 ± 0.1

NA: not assayed; ^a^ Reactions were allowed to proceed for 10 min at 37 °C; ^b^ Reactions were allowed to proceed for 30 min at 37 °C.

**Table 3:** Substrate specificity of NahKs using Glc, Gal, Man, ManNAc, and their derivatives.

Substrates	Percentage Conversion (%)		Substrates	Percentage Conversion (%)	
	NahK_ATCC15697	NahK_ATCC55813		NahK_ATCC15697	NahK_ATCC55813
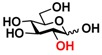 **21** Glc	9.1 ± 0.1	4.7 ± 0.1	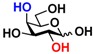 **28** Gal	7.3 ± 0.2	4.4 ± 0.1
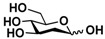 **22** 2-deoxyGlc	44.8 ± 0.2	28.4 ± 0.1	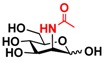 **29** ManNAc	8.9 ± 0.1	5.5 ± 0.1
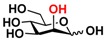 **23** Man	68.0 ± 1.7	37.1 ± 0.4	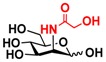 **30** ManNGc	7.6 ± 0.1	5.4 ± 0.2
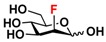 **24** 2F-Man	44.4 ± 0.2	47.0 ± 0.1	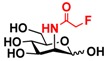 **31** ManNAcF	12.0 ± 0.1	9.1 ± 0.2
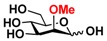 **25** 2Me-Man	9.4 ± 0.5	0	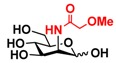 **32** ManNAcOMe	12.0 ± 0.4	7.4 ± 0.3
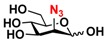 **26** 2N_3_-Man	53.3 ± 0.1	40.2 ± 0.2	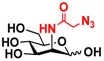 **33** ManNAcN_3_	20.3 ± 0.3	18.6 ± 0.4
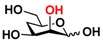 **27** 4-deoxyMan	37.1 ± 0.2	23.9 ± 0.1	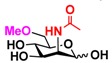 **34** ManNAc6OMe	32.6 ± 0.1	28.9 ± 0.1

The concentration of the enzyme used was 15 μM. Reactions were allowed to proceed for 30 min at 37 °C.

## 3. Experimental

### 3.1. Bacterial Strains, Plasmids, and Materials

Electrocompetent DH5α and chemically competent BL21 (DE3) *E. coli *cells were from Invitrogen (Carlsbad, CA). *Bifidobacterium longum* Reuter ATCC#55813 was from American Type Culture Collection (ATCC, Manassas, VA, USA). Genomic DNA of *Bifidobacterium longum subsp. infantis* (ATCC#15697) was a kind gift from Professor David Mills (University of California, Davis). Vector plasmid pET22b(+) was from Novagen (EMD Biosciences Inc. Madison, WI, USA). Ni^2+^-NTA agarose (nickel–nitrilotriacetic acid agarose), QIAprep spin miniprep kit, and QIAEX II gel extraction kit were from Qiagen (Valencia, CA, USA). Herculase-enhanced DNA polymerase was from Stratagene (La Jolla, CA). T4 DNA ligase and 1 kb DNA ladder were from Promega (Madison, WI, USA). *Nde*I and *Xho*I restriction enzymes were from New England Biolabs Inc. (Beverly, MA, USA). Adenosine-5'-triphosphate disodium salt (ATP), GlcNAc, and GalNAc were from Sigma (St. Louis, MO, USA). GlcNAc, GalNAc, mannose, and ManNAc derivatives were synthesized according to reported procedures [11,12,16,17,18,19,20].

### 3.2. Cloning

NahK_ATCC15697 and NahK_ATCC55813 were each cloned as a C-His_6_-tagged fusion protein in pET22b(+) vector using genomic DNAs of *Bifidobacterium longum* subsp. *infantis* ATCC#15697 and *Bifidobacterium longum* ATCC#55813, respectively, as the template for polymerase chain reactions (PCR). The primers used for NahK_ATCC15697 were: Forward primer 5' ACCCCATATGAACAAC ACCAATGAAGCCCTG 3' (*Nde*I restriction site is underlined) and reverse primer 5' TGAC CTCGAGCTTGGTCGTCTCCATGACGTCG 3' (*Xho*I restriction site is underlined). The primers used for NahK_ATCC55813 were: Forward primer 5' ACCCCATATGACCGAAAGCAATGAAGTTT TATTC 3' (*Nde*I restriction site is underlined) and reverse primer 5' TGACCTCGAGCCTGGCAGC CTCCATGATG 3' (*Xho*I restriction site is underlined). PCR was performed in a 50 μL reaction mixture containing genomic DNA (1 μg), forward and reverse primers (1 μM each), 10 × Herculase buffer (5 μL), dNTP mixture (1 mM), and 5 U (1 μL) of Herculase-enhanced DNA polymerase. The reaction mixture was subjected to 35 cycles of amplification with an annealing temperature of 52 °C. The resulting PCR product was purified and digested with *Nde*I and *Xho*I restriction enzymes. The purified and digested PCR product was ligated with predigested pET22b(+) vector and transformed into electrocompetent *E. coli* DH5α cells. Selected clones were grown for minipreps and characterization by restriction mapping and DNA sequencing performed by Davis Sequencing Facility at the University of California-Davis.

### 3.3. Expression and Purification

Positive plasmids were selected and transformed into BL21(DE3) chemically competent cells. The plasmid-bearing *E. coli* cells were cultured in LB rich medium (10 g/L tryptone, 5 g/L yeast extract, and 10 g/L NaCl) supplied with ampicillin (100 μg/mL). Overexpression of the target protein was achieved by inducing the *E. coli* culture with 0.1 mM of isopropyl-1-thio-β-D-galactopyranoside (IPTG) when the OD_600 nm_ of the culture reaches 0.8–1.0 followed by incubation at 20 °C for 24 h with vigorous shaking at 250 rpm in a C25KC incubator shaker (New Brunswick Scientific, Edison, NJ, USA). To obtain the cell lysate, cells were harvested by centrifuge cell culture at 4000 × g for 2 hrs. The cell pellet was re-suspended in lysis buffer (pH 8.0, 100 mM Tris-HCl containing 0.1% Triton X-100, 20 mL L^−1^ cell culture) containing lysozyme (100 μg/mL) and DNaseI (3 μg/mL). After incubating at 37 °C for 60 min with vigorous shaking (250 rpm), the lysate was collected by centrifugation at 12,000 g for 30 min. His_6_-tagged target proteins were purified from cell lysate using an ÄKTA FPLC system (GE Healthcare, Piscataway, NJ, USA). To do this, the lysate was loaded to a HisTrap^TM^ FF 5 mL column (GE Healthcare) pre-washed and equilibrated with binding buffer (0.5 M NaCl, 20 mM Tris-HCl, pH 7.5). The column was then washed with 8 volumes of binding buffer, 10 volumes of washing buffer (10 mM imidazole, 0.5 M NaCl, 20 mM Tris-HCl, pH 7.5) and eluted with 8 volumes of elute buffer (200 mM imidazole, 0.5 M NaCl, 20 mM Tris-HCl, pH 7.5). Fractions containing the purified enzyme were combined and dialyzed against dialysis buffer (Tris-HCl containing 10% glycerol, pH 7.5, 20 mM) and stored at 4 °C.

### 3.4. Quantification of Purified Protein

Protein concentration was determined in a 96-well plate using a Bicinchoninic Acid (BCA) Protein Assay Kit (Pierce Biotechnology, Rockford, IL, USA) with bovine serum albumin as a protein standard. The absorbance of each sample was measured at 562 nm by a BioTek Synergy^TM^ HT Multi-Mode Microplate Reader.

### 3.5. pH Profile by Capillary Electrophoresis (CE) Assays

Typical enzymatic assays were performed in a 20 μL reaction mixture containing a buffer (200 mM) with a pH in the range of 6.0–11.0, GlcNAc (1 mM), ATP (1 mM), MgCl_2_ (5 mM), and a NahK (0.75 μM). Buffers used were: MES, pH 6.0; Tris-HCl, pH 7.0–9.0; CAPS, pH 10.0–11.0. Reactions were allowed to proceed for 10 min at 37 °C and were stopped by adding 20 μL of cold ethanol to each reaction mixture. Samples were centrifuged and the supernatants were analyzed by a P/ACE^TM^ Capillary Electrophoresis (CE) system equipped with a Photodiode Array (PDA) detector (Beckman Coulter, Inc., Fullerton, CA, USA). CE conditions were as follows: 75 μm i.d. capillary, 25 KV/80 μÅ, 5 s vacuum injections, monitored at 254 nm, the running buffer used was sodium tetraborate (25 mM, pH 10.0).

### 3.6. Effect of MgCl_2_ on the Enzymatic Activity

Different concentrations of MgCl_2_ were used in a Tris-HCl buffer (pH 8.0, 200 mM) containing GlcNAc (1 mM), ATP (1 mM), and a NahK (0.75 μM). Reactions were allowed to proceed for 10 min at 37 °C. Reaction without MgCl_2_ was used as a control.

### 3.7. Substrates Specificity Assays

GlcNAc, GalNAc, and their derivatives (1 mM) were used as substrates in the presence of ATP (1 mM) and MgCl_2_ (5 mM) in a Tris-HCl buffer (pH 8.0, 200 mM) to analyze the substrate specificity of NahKs. Two concentrations (0.75 μM or 15 μM) of each NahK were used and the reactions were allowed to proceed for 10 min (for 0.75 μM NahK) or 30 min (for 15 μM NahK) at 37 °C. For substrate specificity studies of Glc, Gal, mannose, ManNAc, and their derivatives, 15 μM of NahK was used for each reaction and the reactions were carried out at 37 °C for 30 min. All other conditions were the same as for GlcNAc, GalNAc, and their derivatives.

### 3.8. Kinetics by CE Assays

Reactions were carried out in duplicate at 37 °C for 10 min in a total volume of 20 μL in Tris-HCl buffer (200 mM, pH 7.5) containing MgCl_2_ (1 mM), ATP, GlcNAc or GalNAc, and NahK (0.25 μM when GlcNAc and ATP were used as substrates, 0.5 μM when GalNAc and ATP were used as substrates). Apparent kinetic parameters were obtained by varying the ATP concentration from 0.1–5.0 mM (0.1 mM, 0.2 mM, 0.4 mM, 1 mM, 2 mM, and 5 mM) at a fixed concentration of GlcNAc or GalNAc (1 mM), or varying the concentration of GlcNAc or GalNAc (0.1 mM, 0.2 mM, 0.4 mM, 1 mM, 2 mM, and 5 mM) at a fixed concentration of ATP (1 mM) and fitting the data to the Michaelis-Menten equation using Grafit 5.0.

## 4. Conclusions

In summary, two new *N*-acetylhexosamine 1-kinases, NahK_ATCC15697 and NahK_ATCC55813, were successfully cloned. Substrates specificity studies showed that both enzymes are promiscuous and can tolerate various modifications at C2 of GlcNAc and GalNAc. C6-, both C2- and C6-, and both C2- and C3-modified GlcNAc derivatives are also tolerable substrates for both newly cloned NahKs. In addition, both NahKs can use mannose and its C2, C4, and C6 derivatives as substrates. The high expression level (180–185 mg/L culture) and promiscuous substrate specificity of NahKs make them excellent catalysts for application in chemoenzymatic synthesis of carbohydrates.
